# Continuity of long-term follow-up in patients with chronic hepatitis C after sustained virologic response following direct-acting antiviral therapy: a nationwide real-world multicenter cohort study in Japan

**DOI:** 10.1007/s00535-026-02345-0

**Published:** 2026-01-27

**Authors:** Masatsugu Ohara, Ritsuzo Kozuka, Yoshihito Uchida, Chikara Iino, Ryo Sasaki, Hiroki Tojima, Kazuhito Kawata, Satoru Kakizaki, Yoshio Tokumoto, Mizuki Endo, Akira Asai, Jun Inoue, Kenji Nagata, Hirokazu Takahashi, Tetsuro Shimakami, Koji Ogawa, Masaru Enomoto, Tadashi Ikegami, Tatsuya Ide, Naoya Sakamoto, Masaaki Korenaga

**Affiliations:** 1 Department of Gastroenterology and Hepatology, Hokkaido University Graduate School of Medicine, Sapporo, Japan; 2 Department of Hepatology, Graduate School of Medicine, Osaka Metropolitan University, Osaka, Japan; 3 Department of Gastroenterology and Hepatology, Faculty of Medicine, Saitama Medical University, Saitama, Japan; 4 Department of Gastroenterology, Hematology and Clinical Immunology, Hirosaki University Graduate School of Medicine, Hirosaki, Japan; 5 Department of Gastroenterology and Hepatology, Yamaguchi University Graduate School of Medicine, Ube, Japan; 6 Department of Gastroenterology and Hepatology, Gunma University Graduate School of Medicine, Maebashi, Japan; 7 Hepatology Division, Department of Internal Medicine II, Hamamatsu University School of Medicine, Hamamatsu, Japan; 8 Department of Clinical Research, NHO Takasaki General Medical Center, Takasaki, Japan; 9 Department of Gastroenterology and Metabology, Ehime University Graduate School of Medicine, Toon, Japan; 10 Department of Gastroenterology, Oita University, Yufu, Japan; 11 Medical Laboratory, Osaka Medical and Pharmaceutical University, Takatsuki, Japan; 12 Division of Gastroenterology, Tohoku University Graduate School of Medicine, Sendai, Japan; 13 Division of Gastroenterology and Hepatology, Department of Internal Medicine, Faculty of Medicine, University of Miyazaki, Miyazaki, Japan; 14 Division of Metabolism and Endocrinology, Faculty of Medicine, Saga University, Saga, Japan; 15 Department of Gastroenterology, Kanazawa University Graduate School of Medical Science, Kanazawa, Japan; 16 Department of Gastroenterology and Hepatology, NTT Medical Center Sapporo, Sapporo, Japan; 17 Division of Gastroenterology and Hepatology, Tokyo Medical University Ibaraki Medical Center, Ami, Japan; 18 Division of Gastroenterology, Kurume University Medical Center, Kurume, Japan; 19 Hepatitis Information Center, The Research Center for Hepatitis and Immunology, Japan Institute for Health Security, 1-7-1 Konodai, Ichikawa, Chiba Prefecture 272-8516 Japan

**Keywords:** Hepatitis C virus, SVR, Follow-up continuity, Regional core centers

## Abstract

**Background:**

Long-term follow-up is essential after a sustained virologic response (SVR) to direct-acting antivirals (DAAs) in patients with chronic hepatitis C. However, real-world continuity of care and determinants of disengagement are poorly characterized at the national level. Here, we quantified the follow-up continuity within Japan’s government-designated regional core centers and identified independent factors associated with transfer and self-discontinuation.

**Methods:**

We conducted a retrospective multicenter cohort study of 3702 patients with chronic hepatitis C who achieved SVR at 16 regional core centers (2015–2018). Continuation was assessed using Kaplan–Meier analysis and competing-risk analysis, and Fine–Gray regression identified determinants of transfer and discontinuation.

**Results:**

At 5 years, 56% of the patients were followed up, 24% were transferred, and 18% self-discontinued. Older age was significantly associated with transfer (subdistribution hazard ratio [sHR] 1.41, 95% CI 1.23–1.61), whereas hepatocellular carcinoma (HCC) and other malignancies favored continuous follow-up. Self-discontinuation was more frequent with hepatitis C virus (HCV) serotype 2 (sHR 1.36, 95% CI 1.18–1.57) and less common among patients with advanced disease or prior hospitalization.

**Conclusions:**

Within Japan’s core-center network, long-term continuation after SVR is high but not universal. Follow-up was generally maintained for patients with severe comorbidities, while disengagement was more likely among those with lower perceived risk. Strengthening low-intensity, structured support for such patients may improve the continuity and equity of post-SVR care. These findings provide a foundation for optimizing post-SVR care pathways in national liver disease networks.

**Graphical abstract:**

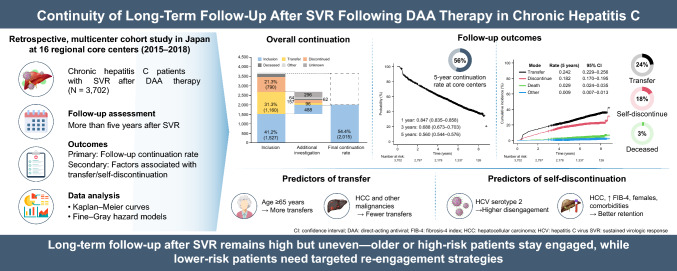

**Supplementary Information:**

The online version contains supplementary material available at 10.1007/s00535-026-02345-0.

## Introduction

Globally, an estimated 50 million people remain infected with hepatitis C virus (HCV), and approximately 240,000 individuals die annually from cirrhosis or hepatocellular carcinoma (HCC) [1]. The advent of direct-acting antivirals (DAAs) has transformed HCV management, achieving viral eradication with high efficacy and safety [2–4], and fueling worldwide efforts toward HCV elimination [5].

To prevent progression to cirrhosis and HCC, it is essential to promote HCV screening and ensure timely linkage to appropriate care for untreated individuals [6–9]. In addition, long-term follow-up, particularly surveillance of HCC after achieving sustained virologic response (SVR), remains crucial for improving patient outcomes [10]. Even after SVR, cirrhosis or HCC may still develop, underscoring the importance of standardized and long-term follow-up strategies [2, 11]. While surveillance recommendations vary internationally [12], continued monitoring has been especially emphasized in Asia [13, 14].

Although numerous studies have reported post-SVR outcomes such as HCC incidence and survival [15–17], the issue of follow-up adherence has received far less attention. Previous reports have been limited to single-center cohorts or registry-based analyses, with a predominant focus on oncologic outcomes rather than continuity of care [15, 18, 19]. Importantly, little is known about how follow-up practices are maintained in structured healthcare systems such as the nationwide network of government-designated regional core centers in Japan.

To the best of our knowledge, no nationwide multicenter study has comprehensively evaluated the follow-up continuation rates after DAA-induced SVR or the demographic and clinical factors associated with disengagement from long-term care. Addressing this gap is critical because viral eradication alone does not guarantee favorable long-term outcomes unless patients remain engaged in appropriate surveillance programs.

In Japan, the government has established regional core centers in each prefecture as central institutions for liver disease management [20]. However, the long-term follow-up status of patients with a SVR in this system has not yet been systematically assessed. Clarifying this issue is essential to understand real-world follow-up practices and develop strategies to ensure sustained patient engagement in care. Therefore, in the present study, we aimed to clarify the follow-up continuation rates among patients who achieved a SVR after DAA therapy at regional core centers across Japan and to identify the demographic and clinical factors associated with continued follow-up in routine practice.

## Methods

### Patients and study design

This retrospective, multicenter cohort study was conducted at 16 government-designated regional core centers for the management of liver disease (regional core centers) across Japan (Supplementary Fig. 1). These centers serve as the central institutions responsible for the coordination and standardization of liver disease care within each prefecture. A total of 72 such centers have been designated nationwide, and the 16 centers included in this study were members of a research group supported by the Ministry of Health, Labour, and Welfare and were able to contribute to clinical data. This study included patients who achieved an SVR after DAA therapy between January 2015 and December 2018. The DAA regimens administered included sofosbuvir/ledipasvir, sofosbuvir/ribavirin, ombitasvir/paritaprevir/ritonavir, elbasvir/grazoprevir, and glecaprevir/pibrentasvir. The DAA regimen was chosen after consultation with the patient and the treating physician. Patients were eligible for inclusion if they achieved SVR and their clinical data were available. Patients were excluded if they had a history of HCC prior to DAA treatment, were enrolled in clinical trials, or developed HCC within 6 months of the end of treatment (i.e., prior to SVR_24_). Patients with formal termination of follow-up at the patient’s request or at the discretion of the treating physician (*n* = 61) were excluded from the primary outcome analyses. However, these patients were included in the descriptive baseline summary (Table 1).

**Table 1:** Baseline characteristics of the patients

Factor	Group	Continued (*n* = 1633)	Transfer (*n* = 1190)	Discontinued (*n* = 818)	Other (*n* = 61)
Age, years		65 (18–89)	68 (16–92)	64 (20–88)	61 (28–81)
Sex, male/female, *n* (%)		713 (43.7)/920 (56.3)	491 (41.3)/699 (58.7)	390 (47.7)/428 (52.3)	25 (41.0)/36 (59.0)
Serotype, *n* (%)	1	1153 (70.6)	788 (66.2)	478 (58.4)	34 (55.7)
	2	466 (28.5)	396 (33.3)	333 (40.7)	26 (42.6)
	Other	14 (0.9)	6 (0.5)	7 (0.9)	1 (1.6)
DAA Regimen, *n* (%)	EBR/GZR	119 (7.3)	78 (6.6)	61 (7.5)	3 (4.9)
	GLE/PIB	262 (16.0)	215 (18.1)	111 (13.6)	13 (21.3)
	OBV/PTV/r	106 (6.5)	76 (6.4)	60 (7.3)	3 (4.9)
	OBV/PTV/r + RBV	3 (0.2)	2 (0.2)	2 (0.2)	0 (0.0)
	SOF/LDV	792 (48.5)	526 (44.2)	327 (40.0)	24 (39.3)
	SOF/RBV	351 (21.5)	293 (24.6)	257 (31.4)	18 (29.5)
Cirrhosis, *n* (%)		313 (19.2)	161 (13.5)	84 (10.3)	4 (6.6)
Platelet count (× 10^4^/μL)		17.0 (1.0–54.4)	17.5 (1.4–42.2)	18.7 (1.4–54.6)	19.6 (3.3–43.0)
AST (U/L)		23 (5–198)	22 (7–438)	22 (3–398)	19.0 (10.0–59.0)
ALT (U/L)		16 (2–258)	15 (4–277)	16 (3–208)	15.0 (3.0–47.0)
Fibrosis-4 index		2.20 (0.24–41.39)	2.18 (0.24–27.05)	2.00 (0.13–23.18)	1.61 (0.38–16.95)
Albumin (g/dL)		4.1 (2.0–5.2)	4.1 (2.1–5.2)	4.1 (2.4–5.1)	4.1 (3.1–5.1)
AFP (ng/mL)		3.5 (0.9–72.1)	3.2 (0.0–42.6)	3.4 (0.7–81.0)	2.6 (1.0–14.8)
Hypertension (%)		379 (23.2)	310 (26.1)	197 (24.1)	17 (27.9)
Diabetes mellitus (%)		201 (12.3)	151 (12.7)	103 (12.6)	10 (16.4)
Dyslipidemia (%)		153 (9.4)	114 (9.6)	59 (7.2)	7 (11.5)

*AFP* alpha-fetoprotein, *AST* aspartate aminotransferase, *ALT* alanine aminotransferase, *EBR/GZR* elbasvir/grazoprevir, *GLE/PIB* glecaprevir/pibrentasvir, *OBV/PTV/r* ombitasvir/paritaprevir/ritonavir, *SOF/LDV* sofosbuvir/ledipasvir, *SOF/RBV* sofosbuvir/ribavirin

Data are presented as medians (range) or numbers (%), as appropriate

Comorbidities such as hypertension, diabetes mellitus, and dyslipidemia were identified based on medical records and were present either before or after DAA therapy. The precise timing of the onset was not uniformly available. Other malignancy refers to incident non-HCC malignancies newly diagnosed after SVR.

### Data collection

Clinical and demographic data collected at baseline, including age, sex, blood test results at the end of treatment (within four weeks of therapy completion), presence or absence of cirrhosis, and HCV serotype (in some patients, the genotype was tested and converted to serotype), were used for classification. For patients who continued follow-up at the regional core centers, additional post-SVR information was collected, including clinical outcomes (occurrence of HCC, development of decompensated liver events, and final clinical status [ongoing follow-up, transfer, discontinuation, or death]) and comorbid conditions (other malignancies, cerebrovascular disease, hypertension, dyslipidemia, and diabetes mellitus). Other malignancy refers exclusively to incident non-HCC malignancies newly diagnosed after SVR during follow-up; pre-existing malignancies before SVR were not included.

For patients who transferred to other institutions, post-transfer information was obtained only from facilities that responded to postal or written inquiries. When available, this included follow-up status at the new institution, occurrence of HCC, development of other malignancies, and cerebrovascular disease. Final clinical status (ongoing follow-up, further transfer, discontinuation, or death) was also recorded.

### Ethics statement

The study adhered to the ethical principles outlined in the Declaration of Helsinki. The Ethics Committee of the Japan Institute for Health Sciences (JIHS, formerly the National Center for Global Health and Medicine) approved the study protocol (approval number: JIHS-G-002541-02). As this was a non-interventional, retrospective, observational study using existing clinical data, an opt-out approach was adopted. All participating institutions publicly disclosed information about the study and provided patients with the opportunity to decline their participation. Inclusion of patients in the analysis was permitted unless they explicitly refused to participate.

### Outcome

The primary outcome was the continuation rate of follow-up after achieving a SVR. The follow-up outcomes were classified as continuation, transfer, or discontinuation. Discontinuation was defined as patients who had scheduled follow-up visits but did not attend and remained unengaged thereafter. Transfer referred to physician-directed referral to another medical institution for ongoing care. Death was not considered a primary follow-up status category in descriptive summaries, but was modeled as a competing event in time-to-event analyses.

The secondary outcome was to identify factors associated with the continuation of follow-up, including demographic, clinical, and laboratory parameters. In addition, we assessed the incidence of HCC and decompensated liver-related events in patients who remained under follow-up at regional core centers and those who were transferred to other institutions.

Outcome ascertainment was performed using electronic medical records at each regional core center. Continuation and self-discontinuation were confirmed by reviewing scheduled visit records and follow-up notes. Transfer was identified based on documented referral to another medical institution, generally initiated by the attending physician, and confirmed by medical records and, when available, written responses from the receiving institutions. Death was ascertained through hospital records or information obtained from transferred institutions.

### Statistical analysis

For time-to-event analyses of follow-up continuity at the regional core centers, time was measured from baseline to the end of follow-up at the core center. In the primary Kaplan–Meier framework, an event was defined as the termination of core-center follow-up (transfer, self-discontinuation, or formal end of follow-up), and death during follow-up was treated as a censoring event. Kaplan–Meier curves were used to depict continuation, and 95% confidence intervals (CIs) were reported at 1, 3, and 5 years. Formal group comparisons were not performed. To decompose exit modes, we estimated cumulative incidence functions (CIFs) for transfer, self-discontinuation, death, and formal end of follow-up using the Aalen–Johansen estimator, and Gray’s test was used for univariable comparisons across strata.

Determinants of exit were assessed with Fine–Gray subdistribution hazard models. Two primary multivariate models were fitted with the events of interest, defined as (1) transfer and (2) self-discontinuation; all other exit modes (including death) were treated as competing events. Candidate covariates (Table 1) were pre-specified on clinical grounds (age, sex, fibrosis-4 index [FIB-4] category, HCV serotype, HCC, other malignancies, diabetes, hypertension, dyslipidemia, prior hospitalization, and decompensated events) and were all entered simultaneously (i.e., without univariable preselection). The results are reported as subdistribution hazard ratios (sHRs) with 95% CIs. Because transfer, self-discontinuation, and death represent mutually competing events for follow-up continuation, Fine–Gray subdistribution hazard models were used to account for competing risks. For sensitivity analyses, we performed multivariate logistic regression models contrasting (1) transfer versus continuation and (2) self-discontinuation versus continuation. Variables with *p* < 0.10 in univariable analyses were entered into the multivariable models. The results are summarized in Supplementary Tables S1 and S2. All *p*-values were two-sided, and statistical significance was set at *p* < 0.05. Analyses were performed using EZR (Saitama Medical Center, Jichi Medical University, Saitama, Japan), a graphical user interface for R.

## Results

### Baseline characteristics of the patients

**Fig. 1:**
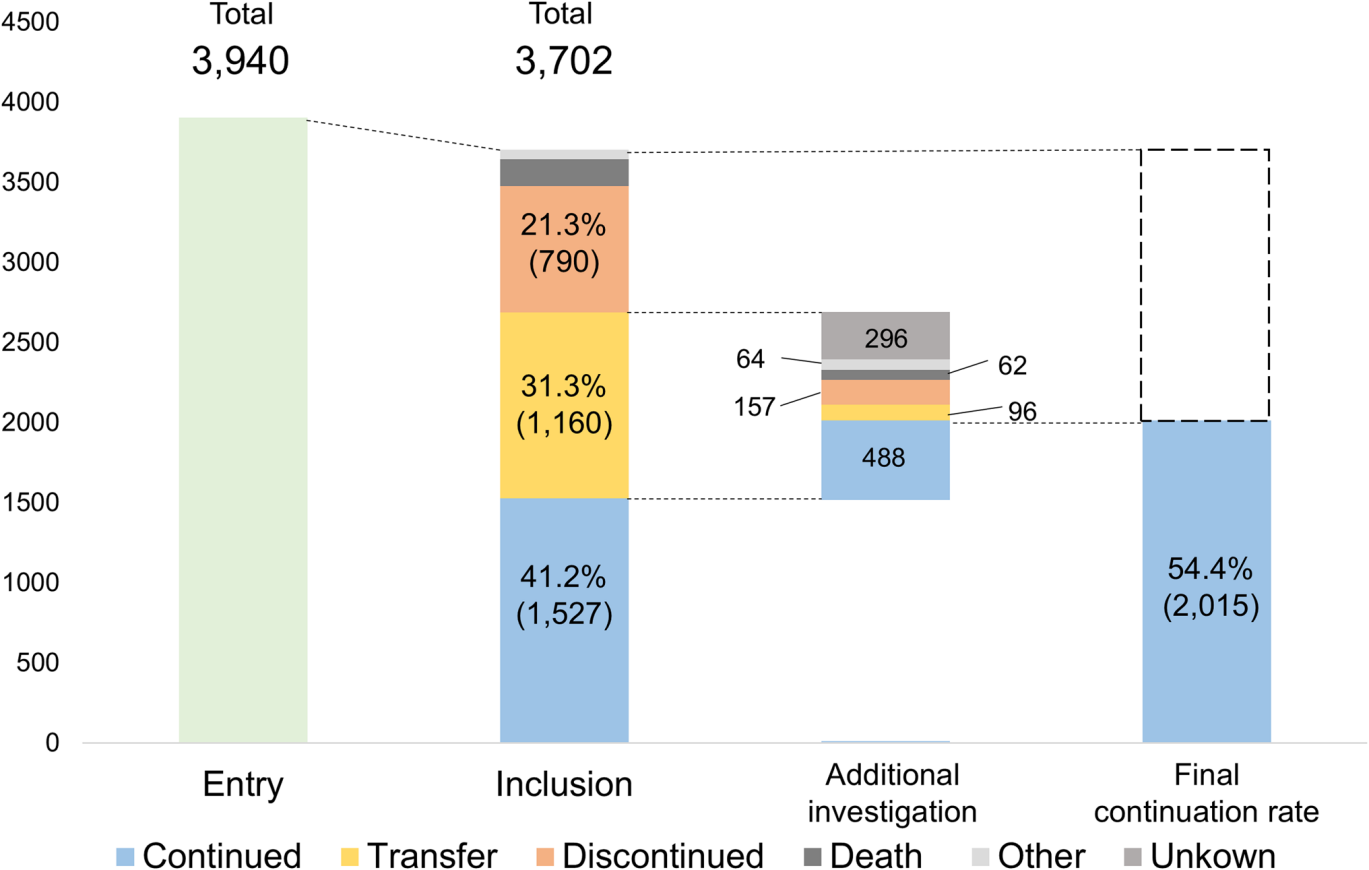
Cohort disposition and ascertainment of follow-up after sustained virologic response (SVR). Of the 3940 screened patients, 3702 were included in this study. At the core centers (“Inclusion”), 41.2% (*n* = 1527) remained in follow-up, 31.3% (*n* = 1160) were transferred, and 21.3% (*n* = 790) self-discontinued. Among the 1160 transferred patients, the post-transfer status ascertained by postal/written inquiry was: continued 488, self-discontinued 157, death 96, and unknown 296 (denominator for these percentages = 1160). The formal end of follow-up (other) occurred in 61 patients. Accordingly, the final follow-up rate at any site was 54.4% (2015/3702), calculated as core center continuation (*n* = 1527) plus continuation confirmed at the receiving institutions (*n* = 488)

Among the 3940 screened patients with chronic hepatitis C who achieved SVR, 3702 were included (Fig. 1). At baseline, the cohort was in their mid-60 s with a slight female predominance (Table 1). At subsequent follow-up, the median age was 65 years in those who remained under follow-up, 68 in those who were transferred, and 64 in those who self-discontinued. The proportion of males was 43.7% in continued follow-up, 41.3% in transfer, and 47.7% in self-discontinuation. HCV serotype 1 was predominant in the continuation and transfer groups (70.6% and 66.2%, respectively), whereas serotype 2 was enriched in the self-discontinuation group (40.7%). Cirrhosis prior to DAA therapy was observed in 19.2% of the continued follow-up group, 13.5% of the transfer group, and 10.3% of the self-discontinuation group.

### Follow-up status

**Fig. 2:**
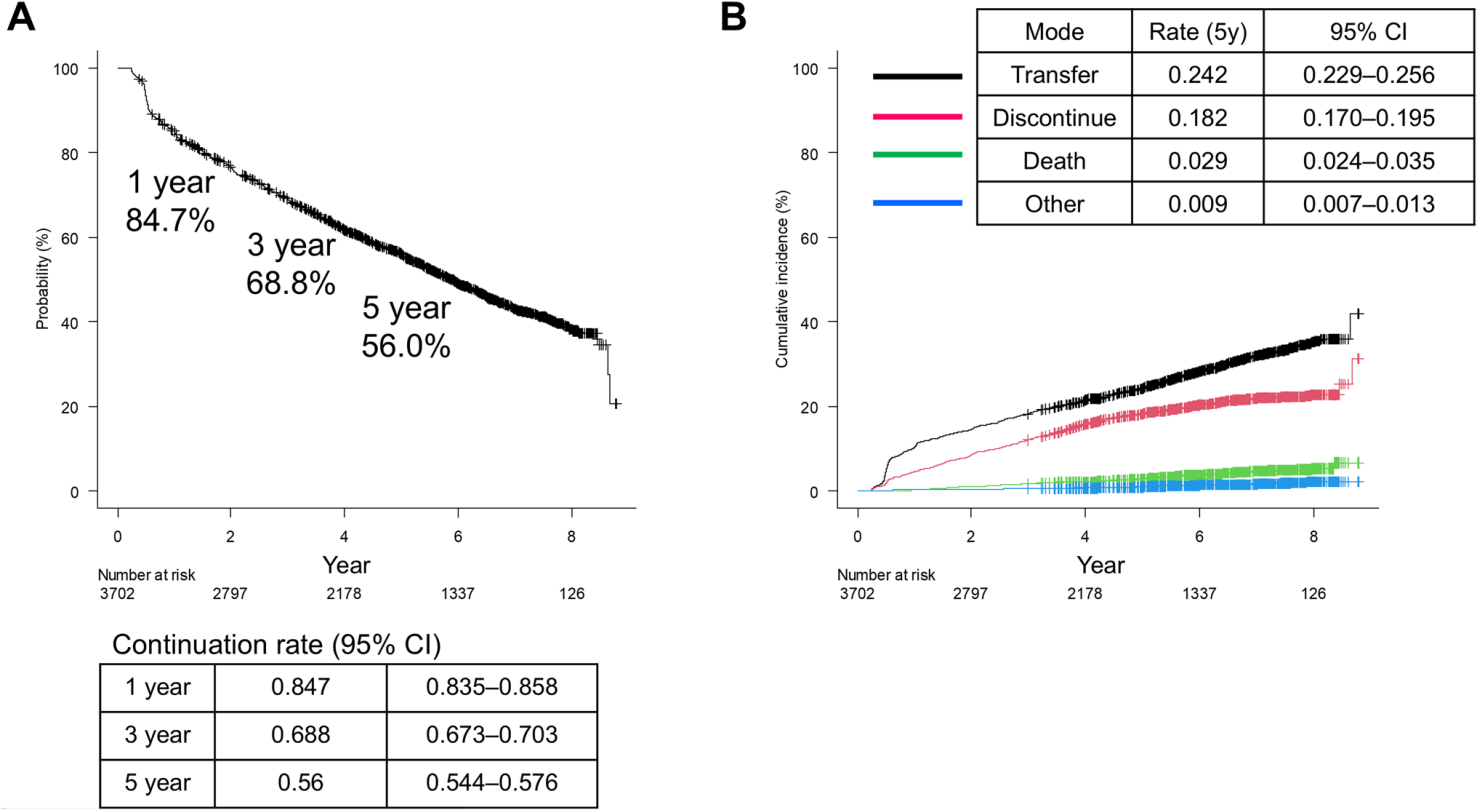
Continuation at regional core centers after SVR: Kaplan–Meier estimates and competing-risk cumulative incidence of exit modes. **A** Kaplan–Meier estimate of continuation at regional core centers after SVR. Event was defined as the end of follow-up at the core center (transfer, self-discontinuation, or formal end of follow-up); death was censored. Estimated continuation was 84.7% (95% CI: 83.5–85.8) at 1 year, 68.8% (95% CI: 67.3–70.3) at 3 years, and 56% (95% CI: 54.4–57.6) at 5 years. Numbers at risk are shown below the *x*-axis. **B** Cumulative incidence functions (CIF) of competing exit modes from regional core centers. Aalen–Johansen estimates for transfer (black), self-discontinuation (magenta), death (green/teal), and formal end of follow-up (blue) after SVR (or SVR_24_). Percentages reported refer to the 5-year time point. At 5 years, the cumulative incidences were 24.2% (95% CI: 22.9–25.6) for transfer, 18.2% (95% CI: 17.0–19.5) for self-discontinuation, 2.9% (95% CI: 2.4–3.5) for death, and 0.9% (95% CI: 0.7–1.3) for formal end of follow-up. Numbers at risk are shown below the x-axis. Note that these fixed-time CIFs may differ from crude proportions because censoring is accounted for. Abbreviations: CI: Confidence interval, SVR: Sustained virologic response

At the time of analysis, 41.2% (*n* = 1527) of patients at the regional core centers remained in follow-up, 31.3% (*n* = 1160) were transferred to other institutions, and 21.3% (*n* = 790) self-discontinued (Fig. 1). Kaplan–Meier estimates showed continuation of 84.7% (95% CI: 83.5–85.8) at 1 year, 68.8% (95% CI: 67.3–70.3) at 3 years, and 56.0% (95% CI: 54.4–57.6) at 5 years (Fig. 2A). Using a competing-risk framework, the 5-year CIF of the exit modes was 24.2% (transfer), 18.2% (self-discontinuation), 2.9% (death), and 0.9% (formal end of follow-up) (Fig. 2B; percentages refer to 5 years). The 5-year Kaplan–Meier continuation (56.0%) was consistent with the complement of CIFs for transfer, self-discontinuation, and formal end at the same horizon.

### Factors associated with transfer (Fine–Gray, event = transfer)

In multivariable subdistribution hazard models (Table 2), age ≥ 65 years was associated with a higher likelihood of transfer (sHR: 1.41, 95% CI: 1.23–1.61, *p* < 0.001), whereas HCC (sHR: 0.37, 95% CI: 0.26–0.54, *p* < 0.001) and other malignancies (sHR: 0.65, 95% CI: 0.48–0.88, *p* = 0.006) were associated with fewer transfers. A higher FIB-4 suggested fewer transfers (≥ 3.25 vs. < 1.3: sHR: 0.85, 95% CI: 0.69–1.03, *p* = 0.100). Sex, HCV serotype, diabetes, hypertension, dyslipidemia, prior hospitalization, and decompensated events were not significant.

**Table 2:** Fine–Gray model for transfer from regional core centers

	sHR	95%CI	*p*-value
Age, ≥ 65 years	1.41	1.23–1.61	< 0.001
Sex, Female	1.06	0.94–1.19	0.370
Serotype			
Serotype 2 (ref.1)	1.00	0.88–1.13	0.990
Serotype other (ref.1)	0.73	0.33–1.64	0.450
Fibrosis-4 index			
1.3–3.25 (ref. < 1.3)	0.94	0.80–1.11	0.470
≥ 3.25 (ref. < 1.3)	0.85	0.69–1.03	0.100
Hypertension	1.08	0.94–1.25	0.260
Diabetes mellitus	0.96	0.80–1.14	0.620
Dyslipidemia	1.03	0.84–1.26	0.770
HCC	0.37	0.26–0.54	< 0.001
Other malignancy	0.65	0.48–0.88	0.006
Decompensated events	0.71	0.41–1.22	0.210
Any hospitalization	0.83	0.67–1.05	0.110

*HCC* hepatocellular carcinoma, *CI* confidence interval, *sHR* subdistribution hazard ratio

Competing events: self-discontinuation, death, and formal end of follow-up

Time origin: SVR (or SVR_24_). Interpretation: sHR > 1 indicates more likely to transfer; sHR < 1 indicates less likely to transfer (i.e., remain at core centers)

### Factors associated with self-discontinuation (Fine–Gray, event = self-discontinuation)

In the complementary model (Table 3), HCV serotype 2 (vs. 1) was associated with a higher risk of self-discontinuation (sHR: 1.36, 95% CI: 1.18–1.57, *p* < 0.001). In contrast, a higher FIB-4 was associated with a lower risk (1.3–3.25 vs. < 1.3: sHR: 0.76, 95% CI: 0.63–0.91, *p* = 0.003; ≥ 3.25 vs. < 1.3: sHR: 0.83, 95% CI: 0.66–1.03, *p* = 0.093). Female (sHR: 0.85, 95% CI: 0.73–0.97, *p* = 0.019) and dyslipidemia (sHR: 0.70, 95% CI: 0.53–0.93, *p* = 0.013) were also associated with reduced risk. Patients with HCC (sHR: 0.19, 95% CI: 0.09–0.38, *p* < 0.001), other malignancies (sHR: 0.49, 95% CI: 0.30–0.79, *p* = 0.003), prior hospitalization (sHR: 0.72, 95% CI: 0.53–0.98, *p* = 0.039), and decompensated events (sHR: 0.33, 95% CI: 0.13–0.87, *p* = 0.024) were less likely to disengage. Age ≥ 65, diabetes, and hypertension were not significant.

**Table 3:** Fine–Gray model for self-discontinuation at regional core centers

	sHR	95%CI	*p*-value
Age, ≥ 65 years	0.88	0.75–1.02	0.100
Sex, female	0.85	0.73–0.97	0.019
Serotype			
Serotype 2 (ref.1)	1.36	1.18–1.57	< 0.001
Serotype other (ref.1)	1.42	0.68–2.98	0.350
Fibrosis-4 index			
1.3–3.25 (ref. < 1.3)	0.76	0.63–0.91	0.003
≥ 3.25 (ref. < 1.3)	0.83	0.66–1.03	0.093
Hypertension	1.14	0.95–1.36	0.160
Diabetes mellitus	1.05	0.83–1.32	0.690
Dyslipidemia	0.70	0.53–0.93	0.013
HCC	0.19	0.09–0.38	< 0.001
Other malignancy	0.49	0.30–0.79	0.003
Decompensated events	0.33	0.13–0.87	0.024
Any hospitalization	0.72	0.53–0.98	0.039

*HCC* hepatocellular carcinoma, *CI* confidence interval, *sHR* subdistribution hazard ratio

Competing events: transfer, death, and formal end of follow-up

Interpretation: sHR > 1 indicates a higher likelihood of disengagement, and sHR < 1 indicates a lower likelihood of disengagement (contributes to retention)

### Supplementary analyses (logistic models and clinical outcomes)

Multivariate logistic regression for the end-of-follow-up status (transfer vs. continued; self-discontinuation vs. continued) yielded directionally consistent associations (Tables S1 and S2). For example, HCV serotype 2 was associated with higher odds of discontinuation (odds ratio [OR]: 1.61, 95% CI: 1.34–1.93, *p* < 0.001), and HCC with lower odds of transfer (OR: 0.27, 95% CI: 0.18–0.41, *p* < 0.001). Additional analyses of clinical outcomes showed a higher mortality and HCC incidence among patients retained at core centers than among those who were transferred or self-discontinued, consistent with the preferential continuation of higher-risk cases (Table S3).

## Discussion

In this nationwide multicenter cohort of 3702 patients with chronic hepatitis C who achieved SVR after DAA therapy, we focused on the long-term continuity of follow-up at government-designated regional core centers. Continuation was high early and declined gradually to 56.0% at 5 years, with most exits attributable to transfer to other institutions (24.2%) or self-discontinuation (18.2%) at 5 years. To the best of our knowledge, these data provide the most comprehensive long-term description of post-SVR follow-up within Japan’s core center network.

Beyond quantities, mode-specific patterns suggest a risk-proportionate organization for care. Older adults were more likely to transfer, whereas patients with HCC, other malignancies, or higher FIB-4 levels were preferentially retained, consistent with the core center mandate to concentrate surveillance on higher-risk populations and with practices in settings where routine post-SVR surveillance is focused on cirrhosis/advanced fibrosis when cost-effectiveness is considered [21, 22]. Conversely, self-discontinuation was more frequent in patients with serotype 2 infections and less frequent among patients with markers of disease severity or healthcare contact (higher FIB-4, female sex, dyslipidemia, prior hospitalization, and decompensation). Previous reports have linked HCV genotype/serotype 2 with dropout around SVR assessment [23], and our findings extend this vulnerability to disengagement in the long-term, routine care period.

Mechanistically, a lower perceived residual risk, fewer symptoms, and transitions to community care may reduce the perceived need for core center visits. These observations argue for low-intensity, structured support, clear post-SVR discharge plans, brief communication of residual HCC risk, coordinated referral, and easy re-entry targeted to groups most prone to disengagement rather than universal intensive surveillance.

Internationally, post-SVR surveillance recommendations differ (e.g., cirrhosis-focused vs. extended to advanced fibrosis vs. broader, network-based approaches) [2, 3, 13, 14]. Across these paradigms, a common challenge is sustaining engagement among patients deemed to be at lower risk. In this context, our findings support a risk-based approach to post-SVR follow-up that is broadly consistent with these international guideline recommendations. Although we did not perform a formal cost-effectiveness analysis, the observed continuation pattern—preferential continuation of patients with HCC, other malignancies, or higher FIB-4 scores, with greater transfer or disengagement among lower-risk profiles—appears consistent with prior cost-effectiveness studies demonstrating that post-SVR HCC surveillance provides the greatest economic value in patients with cirrhosis or advanced fibrosis [21, 22]. In this context, our findings raise the question of whether uniformly intensive follow-up at regional core centers is necessary for all post-SVR patients and underscore the potential importance of risk-based indicators, such as fibrosis severity (e.g., FIB-4) and age, in guiding follow-up strategies. This interpretation should be made cautiously, as our data describe follow-up behavior rather than economic outcomes, and opportunity costs may vary across healthcare systems. A dedicated economic evaluation incorporating Japanese healthcare costs and long-term risk stratification would further clarify the optimal allocation of post-SVR surveillance resources.

Several limitations should be acknowledged. Because this study included patients from 16 of the 72 government-designated regional core centers participating in a specific research network, the generalizability of our findings to all core centers or other healthcare settings may be limited. Post-transfer status was obtained through postal or written inquiries, and incomplete responses may have introduced an ascertainment bias. If all transferred cases with unknown follow-up status were assumed to represent self-discontinuation, the overall proportion of self-discontinuation could range from approximately 25.6% to 33.6%, indicating a potential underestimation in the primary analysis. Self-discontinuation may reflect heterogeneous clinical and behavioral scenarios, including transition to community-based care, patient preference, or unrecognized clinical deterioration, and should therefore be interpreted cautiously as a marker of disengagement from care. Covariates were evaluated at baseline and end of treatment, and temporal changes during follow-up were not modeled. However, time-to-event analyses using Kaplan–Meier and competing-risk methods were applied to appropriately account for follow-up duration and event timing. In the present cohort, we did not identify cases of hepatocellular carcinoma diagnosed after re-engagement following self-discontinuation. However, given the retrospective design and limited post-discontinuation information, such cases may have been unrecognized, and this remains an important topic for future investigation. The strengths include a large sample size, nationwide coverage, and standardized data collection within Japan’s government-designated regional core centers. This network provides a structured and coordinated framework for real-world evaluation of follow-up behavior after SVR, allowing comprehensive assessment under a uniform care system.

In conclusion, continuation after SVR in Japan’s core network is high but not universal. High-risk patients remained under close follow-up, whereas lower-risk patients were more likely to disengage from the study. Lightweight, targeted support for these lower-risk groups may help sustain continuity and equity of long-term post-SVR care.

## Supplementary Information

Below is the link to the electronic supplementary material.

Supplementary file1 Fig. S1 Participating regional core centers for liver disease management in Japan. The 16 participating centers were Hokkaido University, Hirosaki University, Tohoku University, Tokyo Medical University, Ibaraki Medical Center, Gunma University, Saitama Medical University, Hamamatsu University School of Medicine, Kanazawa University, Osaka Metropolitan University, Osaka Medical and Pharmaceutical University, Ehime University, Yamaguchi University, Kurume University, Oita University, University of Miyazaki, and Saga University (TIF 706 KB)

Supplementary file2 (DOCX 32 KB)
