# Supplementary figures and images for: Continuity of long-term follow-up in patients with chronic hepatitis C after sustained virologic response following direct-acting antiviral therapy: a nationwide real-world multicenter cohort study in Japan
Source: J Gastroenterol. 2026 Jan 27;61(4):477–86. doi: 10.1007/s00535-026-02345-0 (PMC13048953; doi:10.1007/s00535-026-02345-0)

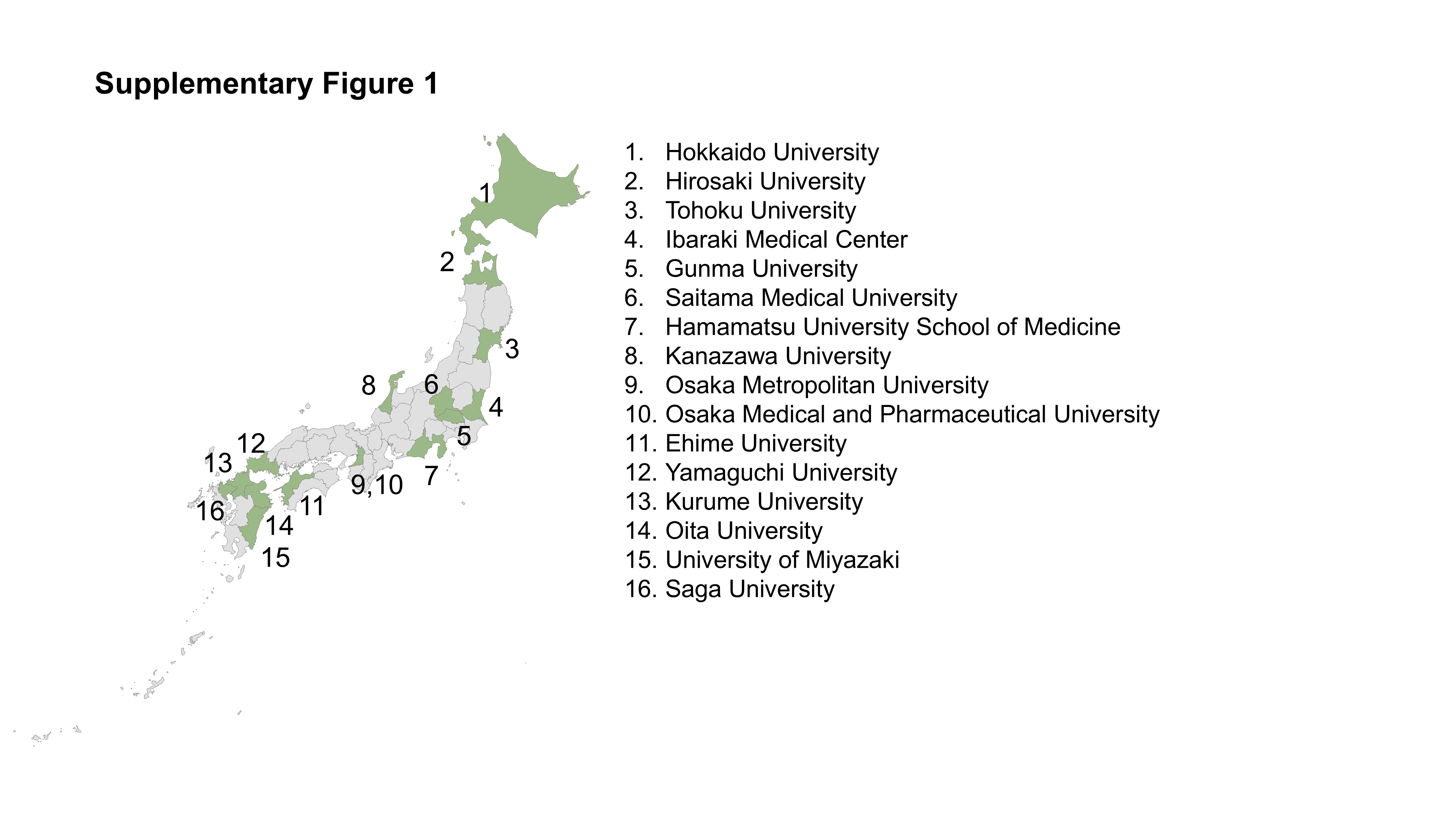

Supplement: Supplementary file 1 — Supplementary file1 Fig. S1 Participating regional core centers for liver disease management in Japan. The 16 participating centers were Hokkaido University, Hirosaki University, Tohoku University, Tokyo Medical University, Ibaraki Medical Center, Gunma University, Saitama Medical University, Hamamatsu University School of Medicine, Kanazawa University, Osaka Metropolitan University, Osaka Medical and Pharmaceutical University, Ehime University, Yamaguchi University, Kurume University, Oita University, University of Miyazaki, and Saga University (TIF 706 KB) [file 535_2026_2345_MOESM1_ESM.tif]
