# Supplementary material for: Continuity of long-term follow-up in patients with chronic hepatitis C after sustained virologic response following direct-acting antiviral therapy: a nationwide real-world multicenter cohort study in Japan
Source: J Gastroenterol. 2026 Jan 27;61(4):477–86. doi: 10.1007/s00535-026-02345-0 (PMC13048953; doi:10.1007/s00535-026-02345-0)
Supplement: Supplementary file 2 — Supplementary file2 (DOCX 32 KB) [file 535_2026_2345_MOESM2_ESM.docx]

**Table S1. Factors associated with discontinuation (reference: continued follow-up)**

|  | Univariate | | | | Multivariate | | | |
| --- | --- | --- | --- | --- | --- | --- | --- | --- |
|  | **OR** | **95%CI** | **P-value** | **OR** | | **95% CI** | **P-value** |  |
| Age, ≥ 65 years | 0.85 | 0.72−1.01 | 0.065 | 1.15 | | 0.95−1.4 | 0.162 |  |
| Female | 0.85 | 0.72−1.01 | 0.060 | 0.84 | | 0.71−1.01 | 0.058 |  |
| Serotype  Serotype 2 (ref. 1)  Serotype other (ref. 1) | 1.72  1.21 | 1.44−2.06  0.48−3.01 | < 0.001  0.688 | 1.61  1.24 | | 1.34−1.93  0.49−3.2 | < 0.001  0.648 |  |
| Cirrhosis | 0.48 | 0.37−0.62 | < 0.001 |  | |  |  |  |
| Fibrosis-4 index  1.3–3.25 (ref. < 1.3)  ≥ 3.25 (ref. < 1.3) | 0.57  0.57 | 0.46−0.71  0.44−0.72 | < 0.001  < 0.001 | 0.63  0.70 | | 0.50−0.79  0.53−0.93 | < 0.001  0.015 |  |
| Hypertension | 1.05 | 0.86−1.28 | 0.63 |  | |  |  |  |
| Diabetes mellitus | 1.03 | 0.80−1.32 | 0.841 |  | |  |  |  |
| Dyslipidemia | 0.75 | 0.55−1.03 | 0.074 | 0.75 | | 0.54−1.03 | 0.074 |  |
| HCC | 0.19 | 0.11−0.32 | < 0.001 | 0.21 | | 0.12−0.36 | < 0.001 |  |
| Other malignancy | 0.53 | 0.34−0.82 | 0.004 | 0.61 | | 0.39−0.96 | 0.034 |  |
| Decompensated events | 0.39 | 0.19−0.81 | 0.011 | 0.58 | | 0.28−1.24 | 0.163 |  |
| Any hospitalization | 0.66 | 0.48−0.92 | 0.014 | 0.73 | | 0.52−1.02 | 0.065 |  |

HCC: Hepatocellular carcinoma, CI: Confidence interval, LC: Liver cirrhosis, OR: Odds ratio.

**Table S2. Factors associated with transfer to other institutions (reference: continued follow-up)**

|  | Univariate | | | | Multivariate | | |  |
| --- | --- | --- | --- | --- | --- | --- | --- | --- |
|  | **OR** | **95%CI** | **P-value** | **OR** | | **95%CI** | **P-value** | |
| Age, ≥ 65 years | 1.35 | 1.16−1.57 | < 0.001 | 1.59 | | 1.33−1.89 | < 0.001 | |
| Female | 1.1 | 0.95−1.28 | 0.203 |  | |  |  | |
| Serotype  Serotype 2 (ref. 1)  Serotype other (ref. 1) | 1.24  0.63 | 1.06−1.46  0.24−1.64 | 0.008  0.341 | 1.24  0.759 | | 1.05−1.46  0.285−2.02 | 0.012  0.581 | |
| Cirrhosis | 0.66 | 0.54−0.81 | < 0.001 |  | |  |  | |
| Fibrosis-4 index  1.3–3.25 (ref. <1.3)  ≥ 3.25 (ref. <1.3) | 0.87  0.78 | 0.71−1.06  0.62−0.99 | 0.156  0.037 | 0.76  0.71 | | 0.61−0.94  0.54−0.92 | 0.012  0.011 | |
| Hypertension | 1.17 | 0.98−1.39 | 0.083 | 1.24 | | 1.04−1.49 | 0.019 | |
| Diabetes mellitus | 1.04 | 0.83−1.3 | 0.763 |  | |  |  | |
| Dyslipidemia | 1.02 | 0.79−1.32 | 0.85 |  | |  |  | |
| HCC | 0.27 | 0.18−0.40 | < 0.001 | 0.27 | | 0.18−0.41 | < 0.001 | |
| Other malignancy | 0.69 | 0.48−0.98 | 0.037 | 0.70 | | 0.48−1.01 | 0.056 | |
| Decompensated events | 0.42 | 0.23−0.77 | < 0.01 | 0.60 | | 0.32−1.12 | 0.109 | |
| Any hospitalization | 0.78 | 0.59−1.02 | 0.071 | 0.80 | | 0.60−1.07 | 0.139 | |

Abbreviations: HCC: Hepatocellular carcinoma, CI: Confidence interval, OR: Odds ratio.

**Table S3. Clinical outcomes during follow-up**

| Factor | Continued  (n = 1633) | Transfer  (n = 1190) | Discontinued  (n = 818) | Other  (n = 61) |
| --- | --- | --- | --- | --- |
| Death, n (%) | 106 (6.5) | 31 (2.6) | 29 (3.5) | 0 (0.0) |
| HCC, n (%) | 149 (9.1) | 45 (3.8) | 15 (1.8) | 1 (1.6) |
| Other malignancy, n (%) | 96 (5.9) | 49 (4.1) | 26 (3.2) | 1 (1.6) |
| Decompensated events, n (%) | 45 (2.8) | 14 (1.2) | 9 (1.1) | 0 (0.0) |
| Non-hepatic hospitalization (e.g., cerebrovascular, pneumonia) (%) | 57 (3.5) | 37 (3.1) | 21 (2.6) | 2 (3.3) |
| Other non-hepatic hospitalization (%) | 99 (6.1) | 59 (5.0) | 33 (4.0) | 1 (1.6) |
| Any hospitalization, n (%) | 149 (9.1) | 86 (7.2) | 51 (6.2) | 3 (4.9) |

Data are presented as the median (range) or number (%), as appropriate. Non-hepatic hospitalization included admissions for cerebrovascular disease, pneumonia, or similar non-liver-related conditions. Other hospitalizations refer to admissions not related to liver disease or the above conditions. Hospitalization was defined as the presence of either of these factors.

The most common types of other malignancies included gastrointestinal cancers (gastric and colorectal), lung cancer, hematological malignancies, breast cancer, and prostate cancer.

Abbreviations: HCC: Hepatocellular carcinoma.
